# Beyond the economic boundaries to account for ecosystem services

**DOI:** 10.1016/j.ecoser.2018.12.007

**Published:** 2019-02

**Authors:** Alessandra La Notte, Sara Vallecillo, Alexandra Marques, Joachim Maes

**Affiliations:** European Commission, Joint Research Centre (JRC), Directorate for Sustainable Resources, Land Resources Unit, Via E. Fermi 2749, I-21027 Ispra, VA, Italy

**Keywords:** Ecosystem services accounting, Supply and use tables, Production boundary, Sustainability assessment, Ecosystem services flow types

## Abstract

Ecosystem services (ES) accounts are essential to quantify and monitor the contribution of ecosystems to human well-being. The System of Environmental and Economic Accounting – Experimental Ecosystem Accounting (SEEA EEA) is the first attempt to provide a set of standards to compile ecosystem accounts. We argue for the inclusion of an ecological perspective in the SEEA EEA that considers ecosystems to be more than input providers to the economy. Ecosystems can act as accounting units capable of producing, consuming and recording changes in regeneration and absorption rates. To account for that we propose (i) to identify ES typologies according to the way in which energy, biomass and information is released to generate services; (ii) to use these typologies to define the concepts of ES potential, ES potential flows, ES demand and ES actual flows; and (iii) to build the ES capacity accounts in monetary terms based on these concepts. These arguments are illustrated with case studies for water purification and crop pollination accounts in European countries. Extending the production boundary would allow the measurement of the sustainable use of ES and the establishment of causality between the use of ES and the value accrued by the economic actors and households.

## Highlights

• An ecological extension of the current SEEA EEA is proposed.
• Broad typologies setting how ecosystem deliver services are identified.
• Concepts of ES potential, ES potential flows, ES demand, ES actual flows are defined.
• Capacity accounts are built based on measurement of ES overuse.
• Examples of water purification, outdoor recreation and crop pollination are shown in EU.

## 1 Introduction

Natural capital accounts include accounting for natural biotic and abiotic resources, and accounting for ecosystems and ecosystem services (ES). The latter family of accounts includes regularly updated measurements of ecosystems and the flows of ES into economic and other human activities. Ecosystem accounting is useful to quantify and monitor the contributions of ecosystems to human well-being (European Commission and European Environment Agency, 2016, p. 106; Mäler et al., 2008, Obst et al., 2016, Wealth Accounting and the Valuation of Ecosystem Service, 2012). The starting point for ecosystem accounting is the System of National Accounts (SNA). The SNA is an international standard for the systematic compilation and presentation of economic data (European Commission et al., 2009). It provides the information needed for economic analysis and policy-making at national level, for example: how much economic sectors produce, how much households consume and save, and the amount of exports and imports with the rest of the world. The SNA represents the entire economy in a simplified way. One of the greatest strengths of the SNA is a robust structure, which ensures consistency across time and across countries while allowing a certain degree of flexibility (European Commission et al., 2009). A demonstration of its flexibility is the development of a set of different environmental accounting structures that are fully compatible and integrated with the SNA (e.g. the System of integrated Environmental and Economic Accounting – Central Framework (SEEA CF)) (United Nations et al., 2014a), SEEA Water, SEEA Energy, and SEEA Agriculture, Forestry and Fisheries). One of these is the System of Environmental and Economic Accounting – Experimental Ecosystem Accounting (SEEA EEA) (United Nations et al., 2014b), which is the focus of this paper.

In its first release, the SEEA EEA and associated technical recommendations (SEEA EEA TR) (United Nations, 2017) have been approached with a strong accounting perspective. The precondition for an integrated accounting system should be consistency with the SNA. Consistency with the SNA guarantees the possibility of comparing SNA economic accounts with ecosystem and ES accounts to illustrate how changes in the economic structure impact the ecosystem and ES and *vice versa* (United Nations et al., 2014a). However, ES are intrinsically different from the goods and services traditionally included in the SNA. This means that in ES, compared with traditional accounting frameworks, there are notions linked to ecology and to spatial analysis that go beyond economics (Daily and Matson, 2008). These disciplines have their own concepts and structures that need to be integrated and made consistent with the underlying SNA structure. Although the accounting mechanism remains the same, ES’s features require some conceptual changes, especially around the economic production boundary. The economic production boundary is the border around what is considered to be production. As a consequence, it determines the units that produce and consume specific services; for example, domestic services produced within the same household (e.g. cleaning, cooking, child care) are excluded by the SNA; consequently households are not considered to be producers of such services and do not consume them. The producer units set the starting point of the production process. In SNA, producer units are referred to as institutional units and are defined as economic entities capable of engaging in economic activities and transactions with other entities (producing and consuming), owning assets and incurring liabilities (accumulating). Production, consumption and accumulation ensure institutional units are regarded as fully active actors in the accounting structure.

As Bartelmus states, accounting for neglected environmental depletion and degradation should be the *raison d'être* of green accounting (Bartelmus, 2015). This implies that ecosystems and ES should not only play the role of input provider to the economic entities but also be measured with respect to production, consumption and changes in their regeneration and absorption rates. For example, inland aquatic ecosystems provide many services, among them water purification. As input provider, we care only about how much pollutant inland waters can remove; as producer unit (institutional unit), we should also monitor the ‘deterioration’ of these ecosystems (as a sort of consumption of ‘fixed capital’). It is not only about quantifying the input the ecosystem provides to human activities (production), but also about the consequences of the interactions with human activities for the ecosystems and their services.

Many studies have recently quantified ES. However, few are consistent with the standards and rules set in the SEEA EEA, with few examples from local (Busch et al., 2012, Remme et al., 2015, Schröter et al., 2014) to continental (La Notte et al., 2017b) scales. In a report that focused on ES accounting at the European scale (La Notte et al., 2017c), some of the challenges that the SEEA EEA has to address in its development process are mentioned: namely to provide the necessary information to assess sustainability, and to establish a causality nexus between ES use and the value accrued by the economic actors.

The hypothesis we propose and test throughout this paper is to upgrade ecosystem units to fully active actors able to produce, consume and record changes in a similar way to what happens for other economic entities that is to consider ecosystem units as institutional units. This possibility was outlined in the SEEA EEA (Annex 6 in UN et al., 2014b): the two hypotheses there described are to consider ecosystems as ‘quasi-sectors’ and to include ecosystem management as an activity of economic sectors responsible for them. In this study, we further develop the first hypothesis. Considering ecosystems as ‘sectors’ implies extending the production boundary by including ecosystem types in the accounting process as ‘producer units’ (institutional units) (Bartelmus, 2014). In this way, relevant concepts from the field of ecology (such as ecosystem service potential) can be properly integrated within the accounting framework. This change has important consequences and implications for the integrated accounting system which we explore and present in this study.

After briefly describing how the context of ecosystem service accounts was developed to show the evolution that has been taking place (Section 2), we structure the methodology section as follows: first, as starting point we frame the role of ecosystems in providing services (Section 3); second, depending on the role of ecosystems, we describe ES flows from an accounting perspective (Section 4); third, based on the typologies of ecosystem service flows, we show how the extended production boundaries would affect the current accounting frame, especially when assessing capacity (Section 5). To illustrate the main arguments discussed in the theory, we introduce some practical examples of ES accounts (Section 6). The discussion (Section 7) highlights how the complementing information can support policy and help in further SEEA EEA developments. The conclusion summarises the main outcomes and foresees the way forward (Section 8).

## 2 Background: the context of integrated accounting systems

The first SNA was finalised in 1953 and the latest SNA was realised in 2008 (European Commission et al., 2009). The SNA includes the flows of goods and services, and the stocks of assets used in the production of goods and services, both measured in monetary terms. The production of goods and services requires inputs from, and has effects on, the natural environment. These effects are the depletion of resources and the generation of wastes returned to the environment. The role of the natural environment in providing resources, absorbing wastes and generally maintaining a habitable world is of crucial importance to economic activity. Any system of economic accounting that omits the environment is thus ignoring a fundamental component of the functioning of the economic system itself. A systematic and structured relationship between the environment and the economy is needed to quantify what the effects of economic activities are on the environment and vice versa, by including the environment in the SNA (Bartelmus et al., 1991, Lange, 1999, Lutz, 1993).

The 1993 SNA (Commission of the European Communities et al., 1993) explicitly included natural resources in its balance sheets and accumulation accounts, as long as institutional units (households, governments, corporations and non-profit organisations) had effective ownership over these assets and drew economic benefits from them.

In 1993, the United Nations Statistics Division (UNSD) published a System of Integrated Environmental and Economic Accounting (SEEA) in a handbook of national accounting (United Nations, 1993), which set out a framework to systematically account for the stocks and flows of environmental resources in a way that was consistent with the SNA. On the one hand, the SNA itself is the core of the SEEA and has been left unchanged. On the other hand, the satellite accounts^1^ of the SEEA supplement the core accounts of the SNA with integrated accounts that expand the asset boundary of national accounts without changing their production boundary. This means that non-produced environmental resources are added to national accounts as external satellite accounts, even if no property right and no economic use is formally set. Both stock and flow accounts were modified, while maintaining consistency with the capital and production accounts of the SNA.

After the publication of the SEEA in 1993, several developing and developed countries started experimenting with the compilation of the SEEA. In 1993, the London Group on Resource Accounting was established as a ‘city group’ with the tasks of defining international best practices for environmental accounting within the SNA and providing a forum for the sharing of national and international expertise in this field. In February 2012, the United Nations Statistical Commission (UNSC) adopted the SEEA Central Framework (CF) as an initial international statistical standard for environmental-economic accounting. The SEEA CF (United Nations et al., 2014a) contains a description of the interactions between the economy and the environment, of stocks and the changes in stocks of environmental assets, and of aggregates and indicators that can be developed. However, the environmental assets in SEEA CF are measured from the perspective of individual natural assets. What is still missing is an ecosystem perspective: if a natural resource is overexploited and disappears, its loss will have consequences not only on the availability of the resource itself, but also on the whole ecosystem that generates the resource and thus on other assets and on human activities. For example, clear-cutting a forest not only implies the loss of wood in the medium and long runs, but also affects carbon sequestration, outdoor recreation, control of soil erosion, mass stabilisation, flood protection, and so on.

To account for natural resource depletion is not enough. There is growing interest from many stakeholders (from the public and private sectors, NGOs, etc.) in the measurement of ecosystems, their likely degradation, and the flow of ES (Science for Environment Policy, 2017). The SEEA EEA Technical Recommendations (United Nations, 2017) are based on the conceptual ecosystem accounting model described in the SEEA EEA, which complements the accounting structures for environmental assets in the SEEA CF; the accounting structures themselves are applications of the principles and structures described in the SNA. The SEEA EEA is composed of different sets of accounting tables, specifically extent and condition accounts that concern ecosystem assets, and supply and use tables that concern ES. The link between the condition account and the supply and use tables is the capacity account. The SEEA EEA also includes several thematic accounts, such as carbon, water and biodiversity. National accounting conventions remain the core of the whole system and determine accounting approaches to the organisation of information.

## 3 Concept: the role of ecosystems in providing services

The ecosystem service concept has, among others, been used to support environmental policy- and decision-making by combining ecological and economic perspectives (an extensive exposition on this topic can be found in TEEB (2010). The ES framework enables us to conceptualise the link between the environment and human activities, to assess the benefits generated by functioning ecosystems to socio-economic systems and, therefore, to design appropriate management policies (Maes et al., 2012). As the SEEA EEA is meant to systematically account for ES, it is important to clarify what exactly is meant by ES within the accounting framework. As reported by Burkhard and Maes (2017), ecosystem properties and conditions provide the ecological basis for ecosystem service potentials. Properties are defined as the structure and processes of an ecosystem; conditions refer to the integrity and health status of an ecosystem. The combination of properties and conditions determines the ability of ecosystems to generate ecosystem potentials to provide services.

There are different theoretical models behind ES. One of the most widely used models in applications concerning ES is the cascade model (Potschin et al., 2016) or variations of that model (TEEB, 2010). The advantage of the cascade model is to link natural systems to elements of human well-being, following a pattern similar to a production chain: from ecological structures and processes generated by ecosystems, to the services and benefits eventually derived by humans. In other words, this framework shows in a simple way how society depends on ecosystems. The disadvantages of this model lie in the facts that (i) complex non-linear and dynamic connections between ecosystem processes and benefits to humans are poorly represented; (ii) it does not include feedbacks; (iii) it appears to follow conventional economic thinking, especially when it comes to the separation between services and benefits. For a critique and extended versions of the cascade model, see Braat and de Groot, 2012, Costanza et al., 2017.

On the one hand, the cascade model helps practitioners to visualise the sequence from ecosystems to humans; on the other hand, it does not adequately represent the complexity of the sequence itself. A focus on the system ecology perspective (La Notte et al., 2017a) identifies services as interactions between and among biotic (i.e. organisms) and abiotic components that generate benefits, which, in turn, lead to a change in human well-being. The purpose of SEEA EEA is to provide relevant information on how economic activity and humans depend on ES and how they may reduce an ecosystem’s capacity to continue generating ES. This kind of information differs from the traditional datasets that feed national accounts and the SEEA CF. It is not about (direct or estimated) measurement of quantities and amounts (mass); it is about ecological processes that, in many cases, are simulated by models that describe how ecosystem units provide flows of services. The SNA accounting structure remains the same in order to keep the linkage with the SNA and SEEA CF. However, some of its concepts need to be extended and changed. Otherwise, no consistent representation of the ecological-economic interaction can be provided. Considering ecosystem types as ‘producer units’ (institutional units) in an enlarged production boundary perspective requires measuring the ecological delivery process before it interacts with economic sectors and households. We need to keep in mind that the advantage of operating in the context of national accounts is that it makes it crystal clear that anything that enters into the economic process is already recorded; for example, once biomass is harvested, any transformation (from transport to processing to delivery to final consumers) is already in the SNA as intermediate and final consumption. What is not recorded in national accounts is the interaction of ecosystem types (as defined in the SEEA EEA; United Nations et al., 2014b) with the ‘demand’ before entering the economic production process. The way this interaction takes place might affect the accounting mechanism that determines how to measure (and eventually represent) overuse and degradation. As current classification systems for ES, such as those of Haines-Young and Potschin, 2018, Millennium Ecosystem Assessment, 2005, TEEB, 2010, consider the purpose of the service, it may be useful to consider an additional aspect and to group ES according to the role of ecosystem types in providing the service. All ES can be characterised according to different typologies of delivery or mediation of matter (more specifically, biomass, energy and information).

We defined five types of ES potential according to the fate of the energy, biomass or information that is produced, absorbed or mediated by ecosystems and, in the last instance, will determine the actual flow of the service used (Table 1).

**Table 1:** Typologies of ecosystem services potential.

Role of the ecosystem	Fate of matter/energy/information	Description	Examples
	Net delivery of biomass or energy eventually leaving the ecosystem	Ecosystems act as sources of matter and energy in the form of biomass. Reference with other classification systems: provisioning services	Generation of mass and biomass
	Delivery of biomass and energy generated within the ecosystem	Ecosystems act as sources of matter and energy by providing suitable habitats. Reference with other classification systems: regulating services (CICES), supporting services (MA) and habitat services (TEEB)	Habitat maintenance, pollination, pest control and disease control
	Matter or energy absorbed by the ecosystem	Ecosystems act as sinks to store, immobilise or absorb matter. Reference with other classification systems: regulating services (CICES and TEEB) and supporting services (MA)	Absorbing pollutants, carbon, nutrients, heat assimilation
	Matter or energy flowing through the ecosystem	Ecosystems act as transformers, changing the magnitude of flows of matter or energy. Reference with other classification systems: regulating services	Water retention, flood control
	Information delivered by the ecosystem	Ecosystems deliver information. The information generated does not modify the original state of the ecosystem. Reference with other classification systems: cultural services	Scenic view, outdoor recreation activities, scientific investigation

Legend: squares represent an ecosystem unit and arrows represent the type of matter/energy/information delivered.

CICES, Common International Classification of Ecosystem Services; MA, Millennium Ecosystem Assessment; TEEB, The Economics of Ecosystems and Biodiversity.

This typology provides a framework for a consistent description of ES flows across disciplines, regardless of the ES classification used.^2^ This study is also essential for a general understanding of the consequences of extending the production boundary (see 4, 6).

Table 1 provides an overview of how ES potential is transformed before becoming an actual flow that is used by people. The process of changing from the potential flow to the actual flow implies an interaction with demand (Jones et al., 2016), since the potential and actual service provision are two different things (Serna-Chavez et al., 2014).

Based on the five types of ecosystem flows, we frame ecosystem types as ‘institutional units’. In fact, production in the SNA excludes natural processes, so, by considering the role of ecosystems in delivering services, we aim to assess production and consumption activities together with their changes in regeneration and absorption rates. Changes in regeneration and absorption rates for some typologies of ES indeed play a role because their current use (recorded in the SEEA EEA) may differ from their sustainable use. The typology shown in Table 1 will now be further explored.

## 4 Ecosystem services as a flow in accounting terms

In the SEEA CF, physical supply and use tables (SUTs) include the environment as an additional column alongside enterprises represented as industries, households and the rest of the world. The environment is not considered an additional type of unit akin to economic units. Rather, the environment is considered a ‘passive’ provider of inputs to the economy and a ‘passive’ recipient of residuals from the economy (United Nations et al., 2014a). An ‘integrated’ accounting system for ecosystems and their services would offer the opportunity to attribute an ‘active’ rather than a passive role to the environment. The SEEA EEA promotes the active role of ecosystems and is open to further important extensions. In the SEEA EEA, there are two models of considering ES in the sequence of accounts (see Annex 6A in United Nations et al., 2014b): model A attributes ES to the ecosystems which are considered as a new additional ‘quasi-sector’ of the economy, whereas model B attributes them to already existing SNA economic sectors considered which can be considered as the managers of these ecosystems; in model B, any adjustment for ecosystem degradation would be attributed to the economic sector and not the ecosystem.^3^ Our proposal considers model A and goes beyond it by giving the ecosystem a fully active role in accounting terms, in a way that is consistent with the SNA.

As stated above, we consider ecosystem types an institutional unit in the same way as for economic units, that is we record natural processes in terms of production and consumption and by recording changes that occur in the unit’s processing ability. This implies an extension of the production boundary that would include not only industries and households but also the ecosystem units. Using ‘external satellite accounts’ provides the opportunity to operate conceptual variations. An important advantage of considering ecosystem types as accounting units in SUTs is the introduction, in the supply table, of information about what ecosystem types are able to offer independently of how much of it will be used. The ecosystem’s ability^4^ to generate services (irrespective of the demand) is what we call here *ecosystem service potential* (or ES potential).

An *actual flow of ecosystem service* (actual flow) is generated when the ES potential interacts with the *ecosystem services demand* (ES demand) and leads to actual use. If there is no interaction with ES demand, there is no actual flow. The actual flow represents the transaction that takes place between ecosystem types and economic sectors and households and is reported in official SUTs. Closely related concepts are the Service Providing Area (SPA) and Service Benefiting Area (SBA) introduced by Burkhard and Kroll (2012), and Service Connecting Areas (SCAs) introduced by Syrbe and Walz (2012).

The concept of SBA corresponds to the ES demand. The way the ES potential interacts with ES demand discloses (through the simple overlay of spatial layers or through ad hoc specific modelling) the spatial relationships (i.e. ‘in-situ’, ‘omnidirectional’, ‘directional’) that are widely applied to assess ES (e.g. by Fisher et al., 2009, Serna-Chavez et al., 2014, Syrbe and Walz, 2012).

The notion of ES potential does not fit all ES. There are in fact ES where regeneration rate (source-provision services in Table 1) and absorption rate (sink services in Table 1) may be affected by excessive use. To measure and account for this, we introduce the concept of *ecosystem services potential flow* (ES potential flow) as the maximum flow of services that the ecosystem type can provide while ensuring its provision through time. Defined as such, the difference between the ES potential flow and the actual flow provides an indication of how sustainably or unsustainably the service is being used. ES potential flow differs from ES potential: the yearly flow of the former can be overused; the latter is only altered when initial conditions change. In the accounting format, the ES potential flow can be reported as complementary information that will not affect the accounting identity between supply and use of actual flow in official tables; the difference between potential flow and actual flow can be reported as an additional mismatch account.

**Fig. 1:**
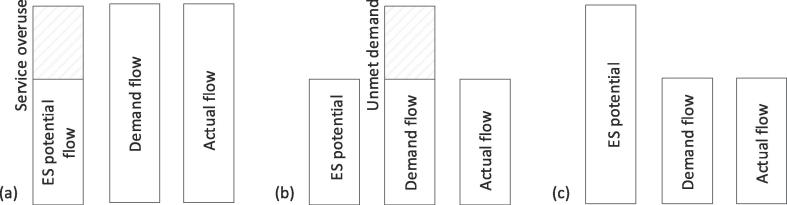
Groups of ES according to the differences in the potential and actual flow. (a) Demand exceeds the ES potential flow and services is overused (e.g. when a resource is extracted above its regeneration rate or when pollutants are emitted above the ecosystem absorption rate). (b) Demand exceeds the ES potential but services cannot be overused (e.g. when people living in a country/region cannot enjoy a range of ES because there are no green spaces). (c) ES potential exceeds demand (e.g. when economic activities are not located where ES that could support them are provided).

For the ES belonging to source-productivity and sink types, it is possible to determine a sustainability threshold by considering the ecosystem type’s regeneration rate and absorption rate. In turn, this sustainability threshold determines the amount of ES potential flow. The overuse of the service occurs when the actual flow is higher than the potential flow and can cause degradation, that is the ecosystem’s capacity to provide the service is decreased (Fig. 1a): the demand would be met at the expense of ES potential flow. Although the link between overuse and degradation is neither direct nor linear in time and space, it is important to measure and record this difference, as it represents degradation over time in ecological terms, and the depreciation of an ecosystem asset in accounting terms.

Regarding sink-related services in particular, as reported in the SEEA EEA (see paragraphs 6.40 and 6.41 of United Nations et al., 2014b), once ecosystem degradation has been assessed, its allocation can be attributed to those economic and human activities that cause degradation (activity-based allocation) or to those that incur the costs of degradation (receiver-based allocation). The first choice requires that the relationship between economic units that cause degradation and the actual flow is set. This is possible when the flow of the service is assessed through biophysical models that simulate biological systems using mathematical formalisations of physical properties and connect human influence (variables of the models) to biological and physical factors and vice versa. Human influence in this case represents the factor that activates the service and that modifies its flow: the enabling actor (La Notte and Marques, 2017).

Source-suitability, buffer and information types (see Table 1) require the ES potential and not the ES potential flow: the actual flow can never be higher than the ES potential delivered by the ecosystem type (Fig. 1b and c). The initial conditions of the ecosystem type (normally related to land cover and land use) determine the amount of ES potential. For these services, the ES potential becomes a flow only when it interacts directly with the ES demand and thus generates the actual flow (Fig. 1b and c).

The ES demand for the service can be higher than the ES potential available, and in this case we can record an unmet demand (Fig. 1b). The definition of unmet demand for ES is already mentioned in other literature, such as by Villa et al. (2014). For example, the protection against the risk of flooding (buffer type) depends on the planning and management practices of the territory, and the ES potential is set by the initial conditions of the accounting period. The actual flow will not influence the initial conditions and will not alter them. If human settlements are not protected against the risk of flooding, there will be an unmet demand. The human settlements demand more buffer capacity from the ecosystem type than the ecosystem type can actually deliver because of its initial conditions.

It can happen that the ES potential is higher than the ES demand (Fig. 1c). For example, in the case of crop pollination, wild pollinators might be present in areas not cultivated with crops that need them (Vallecillo et al., 2018). Spatially explicit information is crucial to identify if met and unmet demands occur and where they are located.

In Table 2, we illustrate how the ES typology can be used in combination with different classification schemes. For this example, we use CICES.^5^ For each ecosystem service in CICES, we identify:

• the associated type as defined in Table 1 – specifically (i) source-productivity, (ii) source-suitability, (iii) sink, (iv) buffer and (v) information;
• the ES potential and ES potential flow;
• the enabling actors that drive the change in the actual flow;
• the meaning of differences between the ES potential, ES potential flow and the actual flow in line with Fig. 1;
• the identification of benefits generated by each ecosystem service flow; they can be considered SNA benefits when there is a direct link with SNA products (this usually happens when the beneficiaries are economic sectors), and non-SNA benefits otherwise (this usually happens when beneficiaries are households);
• the allocation of benefits to beneficiaries;
• the meaning of differences occurring when the supply of benefit differs from the demand – for example, the implications of having unmet demand, especially for non-SNA benefits, which do not appear in accounting systems.

**Table 2:** Reference table for ecosystem services types, enabling actors, benefits and beneficiaries.

Ecosystem services	ES potential and ES potential flow	Enabling actors	When ES potential or ES potential flow ≠ use	Benefit	Beneficiaries	Notes on met and unmet demand
*Source-productivity*						
Agro-biomass growing for crop provision	Level of crop production at which the ecosystem productivity is guaranteed in the long term	Agriculture	Potential flow < use = overuse	Crops (SNA)	Agriculture	To satisfy the demand for crops/fodder/timber/fisheries may cause an overexploitation of natural resources as a consequence of unsustainable practices
Agro-biomass growing for animal husbandry	Level of fodder provision at which the ecosystem productivity is guaranteed in the long term		Potential flow < use = overuse	Fodder (SNA)		
Biomass growing for timber provision	Level of timber provision at which the ecosystem productivity is guaranteed in the long term	Forestry	Potential flow < use = overuse	Wood (SNA)	Forestry	
Biomass maintenance for fish provision	Rate of fish catches at which food webs are maintained over time	Fishery	Potential flow < use = overuse	Fish catch (SNA)	Fishery	

*Source-suitability*						
Crop pollination	Suitability of land to host pollinators in terms of number of species and abundance	Agriculture	If all potential used then maximum efficiency	Pollinator-dependent crops (SNA)	Agriculture	Unmet demand implies less quantity and lower quality for pollinator-dependent crops
Maintenance of nursery population and habitat	Biological and physical support to facilitate the healthy and diverse reproduction of species	Land planning and management practices	If all potential used then maximum efficiency	Conservation of existing conditions in nature (non-SNA)	Global	It is possible to measure unmet demand when a policy target is established
Pest control	Suitability of land to host naturally occurring predators and parasitoids to suppress the populations of pests	Agriculture and forestry	If all potential used then maximum efficiency	Protection against physical damage of crops (SNA)	Agriculture	Unmet demand implies that cultivations are damaged
Disease control	Suitability of land to host naturally occurring predators to suppress vector-borne diseases	Agriculture and forestry	If all potential used then maximum efficiency	Protection against health diseases (non-SNA)	Households	Unmet demand implies that human health is affected

*Sink*						
Air filtration	Amount of pollutants that can be filtered; a sustainability threshold should be applied	Different economic sectors and households (pollutant emitters)	Potential flow < use = overuse	Cleaned air (non-SNA)	Households	Unmet demand implies health effects on population
Water purification	Amount of pollutants that can be diluted/degraded; a sustainability threshold should be applied		Potential flow < use = overuse	Cleaned water withdrawn from water supply company (SNA) from households (non-SNA)	Water supply companies and households	Unmet demand implies higher cleaning costs for water companies and less water for households in case of direct extraction
Soil decontamination	Amount of pollutants that can be decomposed; a sustainability threshold should be applied		Potential flow < use = overuse	Decontaminated soil for production (SNA) and own consumption (non-SNA)	Agriculture and households	Unmet demand implies growing unsafe/unhealthy food
Global climate regulation (marine)	Amount of GHG that can be sequestered; a sustainability threshold should be applied	Different economic sectors and households (GHG emitters)	Potential flow < use = overuse	Mitigation of climate change effects (non-SNA)	Global	It is possible to measure unmet demand when a policy target is established
Global climate regulation (terrestrial)	Amount of GHG that can be sequestered; no sustainability threshold to be applied		Potential = use	Mitigation of climate change effects (non-SNA)	Global	It is possible to measure unmet demand when a policy target is established

*Buffer*						
Mass stabilisation	Ability to prevent/mitigate impacts of avalanches depending on biophysical properties and spatial location.	Land planning and management practices	If all potential used then maximum efficiency	Protection against the risk of avalanches (SNA and non-SNA)	Households and production sites	Unmet demand implies unsafe planning of the territory
Flood protection	Ability to prevent/mitigate impacts of floods depending on biophysical properties and spatial location.		If all potential used then maximum efficiency	Protection against the risk of flooding (SNA and non-SNA)	Households and production sites	Unmet demand implies unsafe planning of the territory
Coastal protection	Ability to prevent/mitigate impacts of coastal erosion depending on biophysical properties and spatial location.		If all potential used then maximum efficiency	Protection against the risk of storms (SNA and non-SNA)	Households and production sites	Unmet demand implies unsafe planning of the territory
Microclimate regulation	Ability to maintain temperature balance at the soil surface		If all potential used then maximum efficiency	Cooling effect (non-SNA)	Households	Unmet demand implies unhealthy planning of the territory
Water retention	Ability to regulate speed and storage of water	Land planning and management practices	If all potential used then maximum efficiency	Aquifer recharge withdrawn by water supply company (SNA) and by households (non-SNA)	Water supply companies and households	Unmet demand implies lacking water storage
Control of erosion risk	Ability of vegetation to control or reduce erosion rates compared with the erosion rates that occur in bare areas		If all potential used then maximum efficiency	Protection against the loss of fertility (SNA and non-SNA)	Agriculture and households	Unmet demand implies the loss of soil fertility

*Information*						
Outdoor recreation	Ability to provide outdoor/nature-based recreation opportunities	Households	If all potential used then maximum efficiency	Increase of tourism (SNA) and components of human well-being (non-SNA)	Tourism sector and households	Unmet demand implies dissatisfaction for population with no daily access to nature-based recreation
Aesthetic beauty	Existence of attractive landscape features	Land planning and management practices	If all potential used then maximum efficiency	Components of human well-being (non-SNA)	Households	Unmet demand implies an unsatisfactory planning of the territory
Source of intellectual investigation and stimulation	Ability to be the source for scientific research and engineering and artistic applications		If all potential used then maximum efficiency	Patents on new discoveries and products (SNA) and components of human well-being (non-SNA)	Professional activities and households	Unmet demand implies the local lack of or the loss for good of some ecological functional and structural features
Education	Ability to be the means and the tools for teaching/learning activities		If all potential used then maximum efficiency	Components of human well-being (non-SNA)	Educational activities and households	Unmet demand implies the lack of some ecological functional and structural features

GHG, greenhouse gas.

The benefit is generated by only the share of the demand that is met which equals the share of the potential that is used (with an effective interaction between ecosystem service potential and demand). In some cases, there is unmet demand, where the service needs are not met.

Table 2 shows two different categories of users: enabling actors (third column) who are the ultimate drivers of changes in the actual flow, and beneficiaries (fifth column) who directly benefit from what is generated by the actual flow. This distinction is useful to understand changes in the service flow and the drivers behind them. Some benefits generated by ES can be quantified as natural resources already reported in the SNA. However, for some other services, benefits are not part of the SNA. In this case, it may be possible to identify some indicators related to human well-being and to build combined presentations to show how ES may affect households in what is not directly reported in the SNA.

When considering enabling actors, it is possible to distinguish between direct actors (when specific economic sectors can be identified) and indirect actors (i.e. land planning and management practices). The latter have economic and/or social drivers that motivate the decisions undertaken by those who administrate the territory.

In Table 2, for ES most closely related to climate change (climate regulation) and conservation of existing conditions in nature (habitat maintenance) the beneficiary is identified as ‘global’, since these issues affect the long-term survival of human beings. These are the cases when a ‘target’ is mentioned: the assumption is that a target is set when there society has a need. A policy target is meant to protect society (in this case, current and future generations). However, this issue needs to be further developed through discussions among policy, accounting and environmental economics experts.

In the previous section, we identified the ecosystem service types that might be relevant for accounting purposes. In the current section, we have explained how the different ecosystem service types may interact with the ES demand to generate the actual flow that is then accounted for in the SUTs: through ES potential or through ES potential flow. In the next section, we check whether or not this setting is affecting the current frame proposed in the SEEA EEA.

## 5 A proposal for extended accounting tables and the calculation of capacity

In this section, we describe how the current accounting tables would be enriched by complementary information as a consequence of an extended production boundary that considers ecosystem types as institutional units.

The previous section describes how (i) for source-productivity and sink services a potential flow can be calculated once a sustainability threshold (referring to regeneration and absorption rates) is established, and the actual use could be higher than, equal to or lower than the potential flow; (ii) for source-suitability, buffer and information services the ES potential can be assessed and the actual use could be equal or lower than the potential, but not higher. The former typology of services requires the addition of further information to the accounting tables. To frame it properly, we need to recall a crucial evolution in the SEEA family of accounts. What in the SEEA CF had been the ‘environment’ satellite accounts now becomes a series of ecosystem types that perform production processes whose outcome is the service provided to human activities (supply table). In turn, human activities affect the consumption, accumulation and future production of services by ecosystem units. In the ‘use’ table, it is possible to allocate the flow of services to their users. The SUTs contain the item ‘accumulation’: in economic accounts, this item is related to the formation of fixed capital and the changes in inventories. Specifically, for economic sectors, fixed assets are produced assets (such as machinery, equipment and buildings) that are continuously used in production over several accounting periods (see Section 1.52 in European Commission et al., 2009). Consumption of fixed capital, or depreciation, is the decline in the current value of the stock of fixed assets as a result of physical deterioration such as wear and tear and obsolescence (see Section 6.240 in European Communities et al., 2009). This implies that there is room in a full accounting system to record positive and negative changes that affect the ability of ecosystem types to provide flows of (individual) ES. For source-productivity and sink services, the threshold (set according to ecological and policy criteria) will allow the ES potential flow to be calculated. We should always keep in mind that we are dealing with flows of ecological processes, not with flows of material assets: the notion of ‘depreciation’ is meant to translate in accounting terms to the concept of ecological degradation due to unsustainable human practices.

The ecosystem capacity is defined as ‘the ability of a given ecosystem asset to sustainably generate a set of ecosystem services into the future’ (United Nations et al., 2014b). It is possible, therefore, to establish a connection between ecosystem service supply and use tables and capacity. We still need to remember a few accounting concepts. As reported in section 6.21 of the SEEA CF, ‘Asset accounts present information on the stock of environmental assets at the beginning and the end of an accounting period and on the changes in the stock over the period. The changes may be of many types […] due to economic activity […] or to natural flows’. Section 6.22 states that ‘changes due to economic activity are recorded consistently in both the asset accounts and the supply and use tables, since extraction represents both a reduction in stock (an asset account entry) and a use of natural inputs (an entry in the physical supply and use table). For environmental assets, this consistency is ensured by defining individual natural resources for the purposes of asset accounting in the same way as natural resource inputs in the physical supply and use table’ (see United Nations et al., 2014a). The SEEA CF applies this definition to natural resources and does not consider ecosystems and ES. However, the same accounting mechanism can be applied to individual ES and the capacity of ecosystem types to provide each individual ES. In the SEEA EEA TR (2017), ecosystem capacity is recognised as central in establishing the connection between ecosystem assets and ecosystem flows, even though the nature of this connection is still not clearly articulated. According to Maes et al. (2018), the focus on ecosystem condition provides useful insights to explore the connection with ES potential in physical terms. In accounting for ES, there is a sequence in biophysical modelling whose outcomes, in some cases, are amounts that correspond to stock (able to provide flows), while in other cases the outcomes correspond to flows. There is also a parallel sequence in monetary terms that consistently accompanies the transformation from the ES potential or ES potential flow into actual flow. Although it is important to highlight the correspondence between the biophysical and monetary dimensions, when dealing with capacity specifically in this section, we explicitly choose to follow the guidelines of SEEA EEA TR and thus consider how capacity can be monetised on the basis of the net present value (NPV) (see chapter 7 of United Nations, 2017).

In the SEEA EEA (United Nations et al., 2014b, United Nations, 2017), reference is made to ‘ecosystem assets’ through an ‘expected basket of ecosystem services’ and the NPV is calculated collectively; we here explicitly disaggregate the ‘basket’ into individual flows of ES and calculate the NPV of each of them. For source-suitability, buffer and information services, the NPV can be calculated from the actual flow as currently suggested in the SEEA EEA. For source-productivity and sink services, the asset account for the institutional sectors ‘ecosystem types’ needs to be accounted differently: interaction with economic sectors and households may generate overuse of the yearly flow of the service (actual flow recorded in SUTs). This overuse could undermine the ability of ecosystem types to provide the same amount of service flow for future accounting periods. In this case, the calculation of NPV should consider if a difference between the potential and actual flow occurs (La Notte et al., 2019). In an extended production boundary, accounting for the potential flow provided by ecosystem types modifies the capacity assessment, where capacity is intended as the critical ecological functioning basis needed to sustain that yearly flow. We should keep in mind that, in this context, accounting is not about mass that can be accumulated and added up but is rather about processes affected by current use or by changes in initial conditions.

**Fig. 2:**
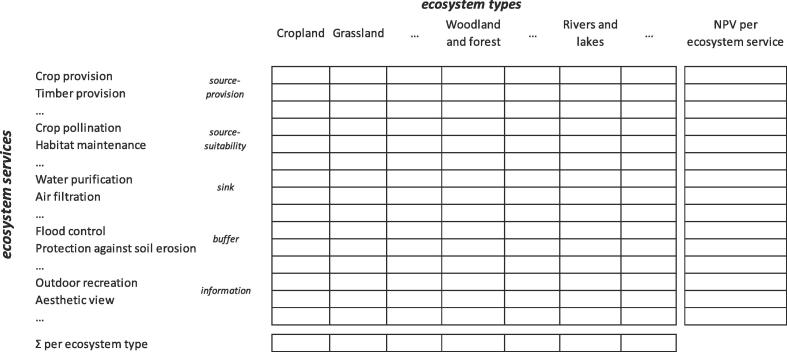
The supply table: linkage between ecosystem services, ecosystem types and capacity as NPV.

The difference with the current approach in SEEA EEA is the modified procedure adopted when dealing with source-provision and sink services (see Table 1) compared with source-suitability, buffer and information services (see Table 1). Accounting for the NPV of individual ES does not contrast with the ideal ‘basket of expected ecosystem services’. When a representative number of ES are assessed and valued, the sum per ecosystem type can be performed as yearly flow or as NPV (see Fig. 2).

Compared with other studies that address the definition of capacity (specifically Hein et al., 2016), the concepts stated here are very similar with only a few differences:

• Hein et al.’s definition of ‘ecosystem service flow’ corresponds to ES actual flow and the ‘potential flow’ corresponds to the ES potential flow as defined for source-provision and sink types (see Table 1).
• The first difference concerns the notion of capacity, which we consider in monetary terms as the NPV. The NPV is closer to the notion of stock than to flow. The actual flow can be higher than the potential flow but cannot be higher than the capacity (unless the ability of the ecosystem to provide a service is totally nullified in a single accounting period). However, the two studies are in agreement regarding what is meant by sustainability and sustainable thresholds.
• The second difference concerns the introduction of ES potential for source-suitability, buffer and information types (see Table 1). When we deal with the delivery of ES, we have two notions: ES potential and ES potential flow. What affects the ES potential are the initial conditions. What affects ES potential flow is the actual use (that can be sustainable or not).

When complementing the SEEA EEA with the SNA and SEEA CF, caution is needed in dealing with already existing environmental and economic accounts, that is, how to combine ‘products’ (SNA) and natural resources (SEEA CF) with SNA benefits and non-SNA benefits (SEEA EEA). Ecosystem services are in some cases an input to SNA products. These are the cases when benefits generated by services may be considered ‘SNA benefits’ because they provide input into production activities; or ‘non-SNA benefits’ when they only support households or society as a whole. Some examples of benefits follow. SNA benefits are flows of environmental assets (e.g. timber, fish, crops) that enter into the production system either as intermediate goods for industries or as final goods for households (when they are directly extracted for own consumption). They can also refer to natural assets affected by quality issues, such as cleaner freshwater and fertile soil (generated by regulating services such as water purification and soil erosion) when used by economic sectors for production; and to risk protection sensitive assets, such as real estate and assets associated with the mitigation of climate change impacts (generated by regulating services such as flood control and carbon sequestration). Non-SNA benefits refer to the flow of all those components that contribute to human well-being, linked to mental and physical health, sense of place and cultural identity, and life satisfaction; they can be both tangible and intangible, and the final beneficiaries are always households.

The importance of specifically reporting benefits in the use table is to clearly separate the service flow generated by ecosystem types from the final benefit received. As shown in Table 2, there might be cases where the enabling actor of the service differs from those who receive the final benefit (beneficiaries). This is especially the case with sink services. In the case of polluters, the enabling actors are those who activate the service and have the power to modify its flow and amount; without them, the service would not be there. One purpose for which we need the accounting tool is to establish the causality nexus between the economic actors’ behaviour and sustainability. We do not deny that the receivers of SNA and non-SNA benefits are those sectors and households who get the outcome of what is generated by the service, but the service itself should be attributed on the principle of activity-based allocation (United Nations et al., 2014b). Examples for water purification are presented by La Notte and Marques (2017).

When the frame clearly identifies services and the generated benefits, overlapping and double counting are more easily avoided, especially for provisioning services. In some cases, it might be enough to account for the benefit generated (SNA or non-SNA) as a proxy for the service; this is possible by complementing the SEEA EEA with the SNA and SEEA CF. The only consistency requirement is that the accounting tables to be filled are the ones related to SNA and non-SNA benefits and not the ones related to services. For example, crop provision (as ecosystem service) differs from actual crops (as SNA product); in a production function perspective, cropland (as ecosystem type) only provides one of the inputs currently used to generate crops. Other inputs may be chemicals, fertilisers, and so on. The contribution of ecosystems in this case needs to be clearly identified and separated from the SNA product: a large quantity of crops (SNA product) does not imply a large contribution of crop provision (ecosystem service) because the main production factors might be human-made (Pèrez-Soba et al., 2015). To guarantee consistency, it would be advisable to create a continuity between the SEEA-AFF (FAO, 2018) and the SEEA EEA, especially for what concerns the primary sector products. An example of how to disentangle the ecosystem’s contribution from the final benefit is provided, for crop pollination, by Vallecillo et al. (2018).

In the next section, a few examples, based on initial applications on water purification and crop-pollination at EU level, translate these conceptual accounting notions into practical terms.

## 6 Illustration of key messages through ecosystem services accounts

In this section, we illustrate the main argument presented in this paper with two examples. Enlargement of the production boundary at the basis of ES accounting should consider ecosystem types as institutional sectors that interact with the ES demand and generate ES actual flow. In practical terms, this would be translated into the drafting of extended accounting tables, presenting complementary information to the SUTs that are needed for a consistent natural capital accounting. We illustrate two different types of extended accounting tables depending on the typology of ES described in Table 1. The first example is for crop pollination (source suitability service) (see Section 6.1) (Vallecillo et al., 2018). This example illustrates those ES for which the actual flow is generated by the interaction between ES potential and demand. This interaction may also generate a mismatch between ES potential and demand (see Fig. 1b and c), which could be used as complementary information to be reported in the extended accounting tables. This is the case for source suitability, buffer and information services (Table 1). The second example is for water purification (sink service) (see Section 6.2) (La Notte et al., 2017b) and illustrates those ES for which the potential ES flow needs to be assessed based on a sustainability threshold. A sustainability threshold is used to define the potential flow that, if equal to or above the actual flow, guarantees a sustainable regime; however, if the potential flow is below the actual flow (Fig. 1a) it leads to overuse and eventually degradation. This is the case for source-productivity and sink services (Table 1). The extended accounting tables reporting potential flow are not only complementary information for the SUTs, but also input data for the calculation of capacity in monetary terms.

### 6.1 Mismatch between ES potential and demand: crop pollination accounts

**Fig. 3:**
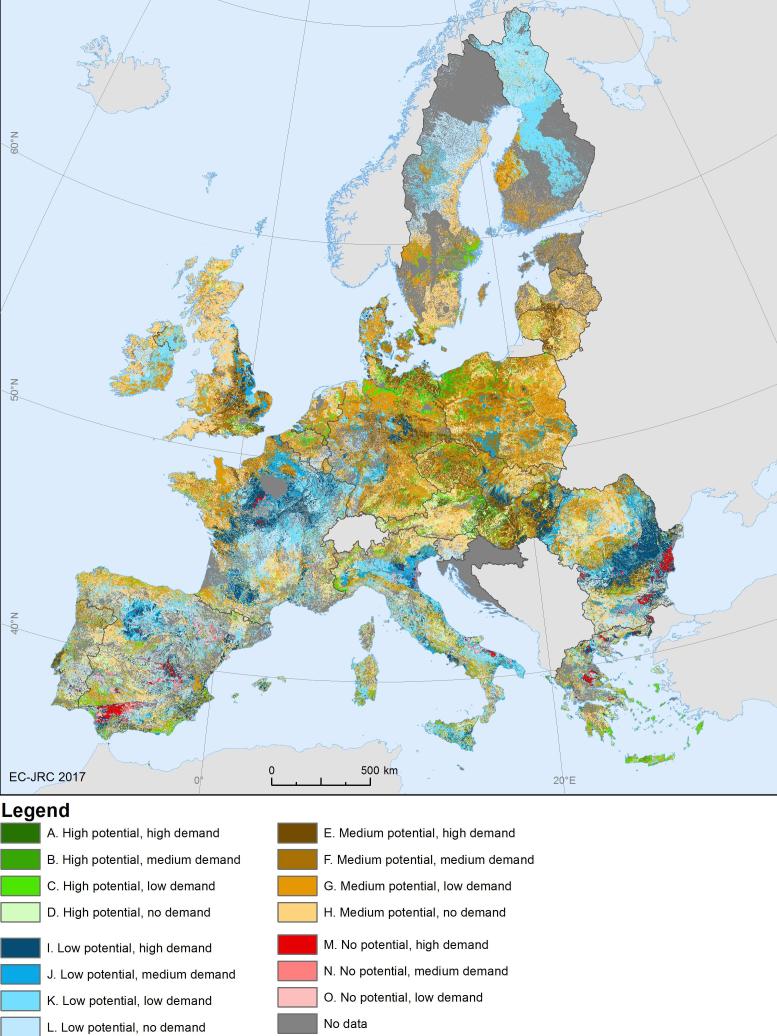
Map of mismatch between ES potential and demand for crop pollination in 2006 (source: Vallecillo et al. (2018)).

The first example reported here concerns a regulating ES characterised by being a source-suitability service (see Table 1). Crop pollination as ES is the fertilisation of crops by insects that maintains or increases crop production. The example we show here considers specifically wild pollinators and integrates two different models (see Vallecillo et al., 2018 for further details): an expert-based model for solitary bees (computed with the ESTIMAP toolbox, Zulian et al., 2013b) and a species distribution model for bumblebees (Polce et al., 2013), predicted with observed species records. Both models quantify the ES potential and are based on land cover, climate data and the distance to semi-natural areas. The demand for crop pollination was quantified as the extent of pollinator-dependent crops, following the methodology described by Zulian et al. (2013a). We used the spatial data derived from the Common Agricultural Policy Regional Impact analysis (CAPRI) model (Britz and Witzke, 2014, Leip et al., 2008) to quantify the demand as the number of hectares per square kilometre. We considered 10 crop types that benefit from insect pollination to different extents. The overlap between the pollination potential and the demand for pollination is used to quantify the area generating the actual flow of service^6^. There might be areas – where the crop extent is covered by low (Fig. 3, ‘I. Low potential, high demand’) and no pollination potential (Fig. 3, 'M. No potential, high demand’) – that have been considered in terms of ‘unmet demand’ in the accounting exercise. On the other hand, there might be areas where pollination potential (Fig. 3, ‘C. High potential, low demand’, ‘D. High potential, no demand’; and ‘G. Medium potential, low demand’, ‘H. Medium potential, no demand’) exists but it is not used because there are reduced/small extent of pollinator-dependent crops.

**Fig. 4:**
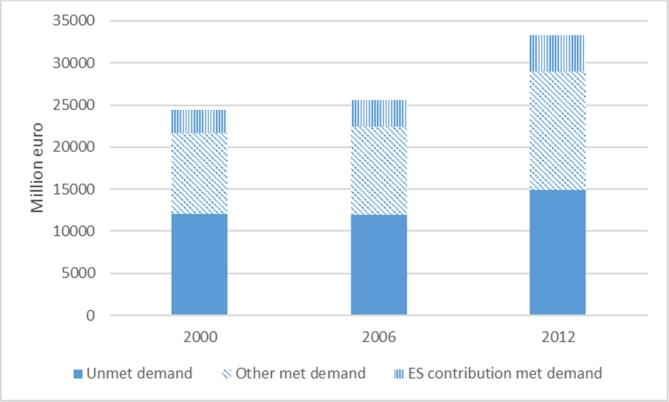
Monetary assessment of crop pollination service and its role with respect to pollination-dependent crops for the EU, in millions of euros (source: data processed from Vallecillo et al. (2018)).

The actual flow of crop pollination is quantified as the yield production attributable to pollination according to the level of pollinator-dependency of different crop types. This procedure involves the calculation of a ratio for pollination contribution that depends on a biophysical assessment (Vallecillo et al., 2018). This procedure allows the ecosystem contribution (service flow) to be disentangled from the SNA products (asset flow). Moreover, within the total production (reported in the SNA) it is possible to identify different components: (1) the proportion of the production that is derived from the ‘met demand’, which includes the ecosystem contribution and the rest of the production not attributable to the ES (related to the dependency ratio), and (2) The proportion of the production generated in areas of ‘unmet demand’, where the pollination potential may not be high enough to provide the service. Fig. 4 reports the crop production in monetary terms of pollinator-dependent crops, with reference to met demand, which includes the ecosystem contribution, and unmet demand. Within ‘met demand’ we disentangle the ecosystem service from SNA products. The contribution of cropland in terms of pollination is disentangled from the total economic aggregate reported in agricultural statistics, by using the pollination contribution coefficients calculated through the biophysical model.

Fig. 4 shows that a remarkable part of SNA pollination-dependent production does not benefit from pollination. This is important information for policy makers: the unmet demand of crop-pollination highlights that there is room to enhance crop pollination, and therefore the natural capital. This could generate (i) higher production and/or (ii) more sustainable production practices in countries where pollinator-dependent crops do not receive enough crop pollination service. To invest in creating habitats that are conducive for crop-pollination could (i) increase crop production and/or (ii) reduce the human factors (especially chemical fertilisers) in the production process by keeping the same amount of production. The two options vary according to the characteristics of different areas and to the current management practices currently in place. The spatially explicit assessment of ES potential and actual flow is essential for this kind of analysis.

### 6.2 Differences between ES potential flow and actual flow: water purification accounts

The example reported here concerns a regulating ES characterised by being a sink service (see Table 1). The case study assesses the water purification services that take place in inland waterbodies in Europe. Excessive nitrogen loading causes water pollution, both in Europe and globally; nitrogen (N) is thus used as a proxy for water quality. The biophysical model (Grizzetti et al., 2012) contains a spatial description of nitrogen sources and physical characteristics that influence nitrogen retention. The area of study is divided into a number of sub-catchments that are connected according to the river network structure. For each sub-catchment, the model considers the input, both of nutrient diffuse sources (e.g. mineral fertilisers and manure applications) and of point sources (e.g. industrial and waste water treatment discharges). Diffuse sources are reduced, both by the processes occurring in the land (e.g. crop uptake, denitrification and soil storage), and those occurring in the aquatic system (e.g. aquatic plant and microorganism uptake, sedimentation and denitrification), while point sources are considered to reach the surface waters directly and are therefore affected only by the river retention. We use modelled nitrogen retention in water bodies as indicators of the actual flow of the water purification service. However, we should consider that there is a threshold in the nitrogen concentration of surface water below which the removal of nitrogen by the different ecological processes is sustainable from an ecosystem point of view. The tentative threshold concentration applied for this specific case study is of 1 mg N l^−1^ (Maes et al., 2012), which corresponds to the level at which water bodies start eutrophication. Using data on average river flow in combination with the critical nitrogen concentration allows us to calculate the critical threshold below which no environmental damage is expected. Increases in nitrogen loading far above the critical loading level will result in the degradation of aquatic ecosystems. Further details on the biophysical assessment and the full methodology for the monetary valuation of this ES are described in previous publications (La Notte et al., 2017b, La Notte et al., 2012). Here the focus is on accounting. Specifically, it is important to show how supply and use tables can be enriched when complemented with the additional information provided when accounting for potential flow.

**Fig. 5:**
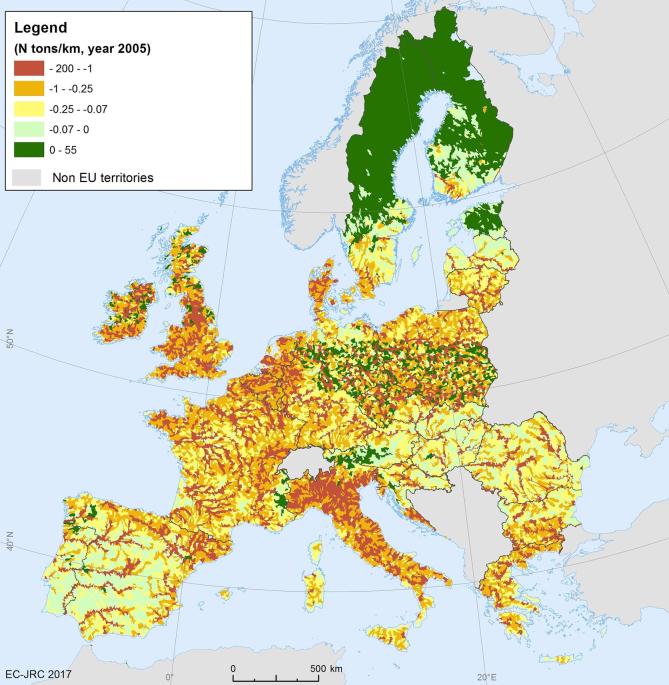
Map of mismatch between ES potential flow and ES actual flow for water purification in 2005 (source: data processed from La Notte et al. (2017b)).

Supply and use tables as currently proposed in the SEEA EEA report ES actual flow. An extension of the production boundary would allow the addition of complementary information concerning ES potential flow. The differences between potential and actual flow (see Fig. 5) show if water purification is being overused and where the overuse takes place (i.e. where actual flow is higher than potential flow).

**Fig. 6:**
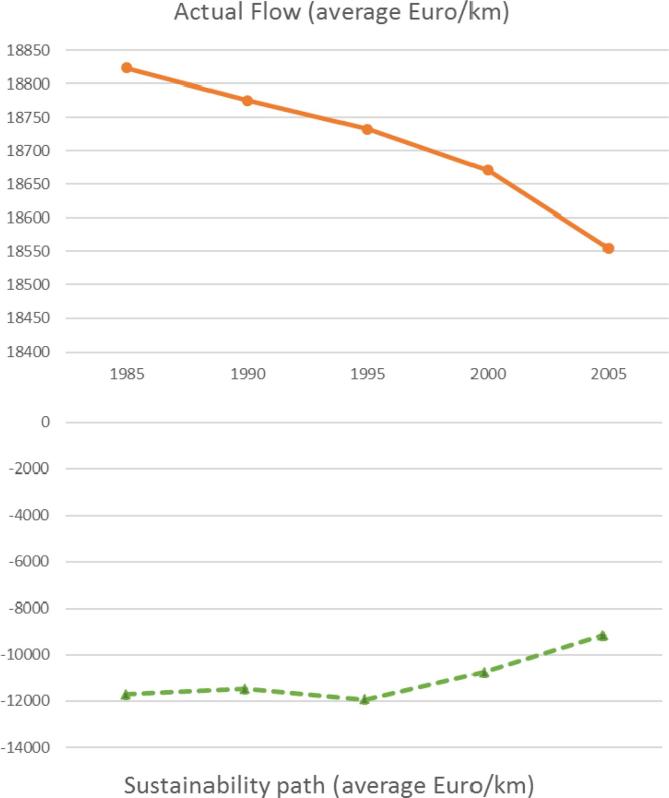
Trend in the actual flow of water purification and the increase in the sustainability path (1985–2005) (source: data processed from La Notte et al. (2017b)).

While the difference between potential and actual flow shows an overall pattern of unsustainable use (Fig. 5), the evolution over time (Fig. 6) shows a reduction in the overuse of the service (i.e. a decreased difference between the potential and actual flow). This difference is illustrated in Fig. 6 as the sustainability path.

This implies that the use of the service is becoming less unsustainable. The decrease in the value of water purification actual flow is not a negative message for policy makers and the general public because it implies an improvement in the sustainability path (Fig. 4). The economic figure that should be reported to support a successful policy is the increase in value of the potential flow.

**Fig. 7:**
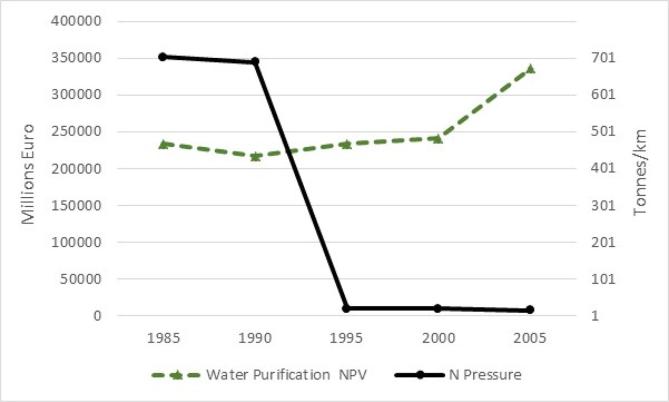
Trend of water purification capacity (total million euros – primary axis) and nitrogen emissions (tons/km – secondary axis) from 1985 to 2005 (source: data processed from La Notte et al. (2017)).

Based on the possibility of assessing the potential flow, water purification accounts provide a good case to calculate capacity in monetary terms. We can expect that if we calculate the NPV from the actual flow we will get a decreasing capacity from 1985 until 2005 (see Fig. 6). On the other hand, if we calculate the NPV from the potential flow we will get an increasing capacity from 1985 to 2005. We could easily confront the latter (expressed in millions of euros) with the tonnes of nitrogen per kilometre of river length that constitute the driving pressure in the biophysical model we used for the water purification assessment (Fig. 7).

The remarkable decrease of nitrogen shown in the graph in relative terms (tons/km) is most likely caused by the Nitrates Directive.^7^ The policy directive is thus succeeding in putting a break in the degradation of freshwater ecosystems. However, we need to keep in mind that Fig. 7 shows the value of natural capital only for inland waters and only for water purification. Fig. 7 also points out that there is a time lag between policy implementation and the impact on water purification capacity. The important change, compared with the current SEEA EEA guidelines, is to consider ES potential flow rather than ES actual flow in the calculation of capacity in monetary terms. This principle applies to source-production and sink services.

## 7 Discussion

Following the evolution of integrated accounting systems, the SEEA EEA represents an excellent starting point to address the issues of how to account for ES in a way that remains consistent with the SNA structure. It provides a picture of the actual situation, and without this solid basis no progress can be made in this field of applied research. In this paper, we aim to remain linked to the main frame (SEEA EEA) and to provide additional information. To do that, we propose to act on the SNA production boundary and specifically on the role of ecosystem types as institutional units. As shown in the case studies, considering ecosystem types as ‘full accounting units’ (by extension of the production boundary) does generate a remarkable difference for the whole frame. In the current frame, ecosystem types as ‘additional inputs’ generate flows that *depend on* the economic production and consumption; in the extended frame, treating ecosystem types as ‘full accounting units’ implies that their action and interaction with economic sectors and households is recorded *together with* economic units. The latter perspective means that natural processes are integrated in national accounts through the inclusion of ES potential flow in assessing the capacity. The identification of the services that needs the assessment of ES potential flow as complementary information requires us to consider the role of ecosystems in delivering services: the well-known classification into provisioning, regulating and maintenance, and cultural services is not enough. Regulating services can include services that require the assessment of the potential flow (sink) and services that do not (source-suitability, buffer). Once identified and assessed, the inclusion of natural processes in national accounts allows us to track sustainability and to establish meaningful linkages (causal relationships) with the economic sphere. The availability of measurements for potential flow can also facilitate the linkage with the ecosystem condition account. As the example of water purification (see Section 6.2) shows, the potential flow is strictly linked to the ecosystem condition: it depends on it, and its progressive degradation will eventually affect the ecosystem condition. It would be useful to track the causes of degradation per ecosystem service and per economic sector. Contrariwise, when the ecosystem condition improves, it would be useful to allocate which ES contribute to it the most and whether or not sustainable practices applied within the enabling economic sectors and/or households were successful. Policy analysis can benefit greatly from this additional information, structured according to the accounting rules, and thus able to be integrated with economic accounts for further, deeper analysis.

## 8 Conclusions

There are two features that distinguish SEEA from other systems and tools: the integration of environmental data with economic accounts; and the comprehensive treatment of all important natural resources, linking them to the economic sectors that rely on them, directly and indirectly, and those sectors that affect them. The availability of SEEA should enable governments to set priorities, to monitor the environmental impact of economic policies more precisely, to enact more effective environmental regulation and resource management strategies, and to design more efficient market instruments for environmental policies (FAO, 2003). This is the final purpose of an integrated accounting system: to provide enhanced information for policy uses. The rigour of the SNA structure and accounting rules and at the same time the flexibility to expand existing data or to add new data allow experimentation on how to provide enhanced information.

In this paper, we have discussed the integration of the natural process into the accounting mechanism. We pursued this integration ambition through:

• the identification of broad typologies that set how ecosystems deliver services (the five types reported in Table 1);
• the analysis of how the interaction between what ecosystem service deliver and the demand generates the actual flow (concepts of: ES potential, ES demand and ES potential flow); and
• the integration of changes in regeneration (source-provision services) and absorption (sink services) rates through ES potential flow into the accounting frame (calculation of capacity expressed in monetary terms as NPV).

Compared to the current SEEA EEA, only source-provision and sink types need some modification (i.e. potential flow) to be introduced in the overall accounting framework. However, these two ecosystem service types are the ones subject to annual modification (resource extraction and emission of pollutants) and are those that could more quickly lead to overuse and eventually degradation. For other ES, major changes might take place over a long time (because they concern land use), and actual annual use does not affect them. It therefore remains important to extend the production boundary and include the natural process (specifically accounting for regeneration and absorption) in the accounting mechanism.

Overall, the first phase in developing ecosystem accounts had been led (as it had to be) by the rigorousness of the SNA accounting frame. The same SNA production boundary and the same SNA accounting mechanism are applied in order to reach a coherent overall structure. Since the introduction of external satellite accounts offers the possibility to experiment with new concepts, and since ecosystem-related sciences require concepts that differ from pure mainstream economics, this paper proposes (in the second phase) an ecological integration toward the second phase of SEEA EEA evolution.

We here consider the support that ES accounting could provide to preventive action rather than remedial actions: the assessment of sustainability becomes the key of preventive action, as signals are provided to policy makers if a situation is becoming critical or whether any policies prove to be successful or not.

This is the motivating factor for proposing additional information to complement the SEEA EEA in order to systematically and consistently quantify information on the degradation of ES and their causal linkage with human activities. Still, more applications have to be undertaken to reach a mature and solid version of this framework because each ecosystem service has its own peculiarity. Moreover, we should not forget that integrated ecological-economic accounting framing is a human construct that can be built, developed and improved step by step. The future third step of SEEA EEA evolution will probably be on the assessment of human well-being derived from ES. Jumping to ES-dependent human well-being without properly framing the ecological step is not possible and the attempt could lead to oversimplification and misleading messages.

## Footnotes

1 Satellite accounts are additional accounts that follow the same structure and rules as SNA accounts.

2 CICES is the classification system we refer to regarding Table 1. The reasons are that CICES is specifically mentioned in the SEEA EEA TR (UN, 2017), and it has been adopted for the MAES initiative (Mapping and Assessment of Ecosystem Services) in Europe (Maes et al., 2012).

3 This assumption implies that the institutional sector (economic unit) that manages the ecosystem is responsible for the generation of ecosystem services. When explained in the SEEA EEA, this assumption is defined as weak, and we agree with this statement.

4 This is what it is usually quantified in ecosystem service assessment with dimensionless indicators (from high to low ecosystem ability).

5 The current classification is drawn from the material available in www.cices.eu. However, a few changes have been made: (i) in the case of provisioning services, the ecological production function is isolated from the benefit, to avoid ambiguities; (ii) a few ecosystem services related to physical and chemical conditions (which in the SEEA EEA accounting scheme belong to the ecosystem condition account) were not considered; (iii) cultural services are considered as long as their measurability mostly links to ecological features (in fact, for some CICES cultural services, it seems more appropriate to use other social value based approaches).

6 Actual flow can be assessed for only 25 Member States for which data on demand were available.

7 To protect water quality from agricultural sources polluting ground and surface waters, the Nitrates Directive was issued in 1991 as part of the Water Framework Directive. It represents one of the main instruments for the protection of waters against agricultural pressures. Ref. http://ec.europa.eu/environment/water/water-nitrates/index_en.html.

## References

[b0005] Bartelmus P. (2014). Environmental-economic accounting: progress and digression in the SEEA revisions. Rev. Income Wealth.

[b0010] Bartelmus P. (2015). Do we need ecosystem accounts?. Ecol. Econ..

[b0015] Bartelmus P., Stahmer C., Tongeren J.v. (1991). Integrated environmental and economic accounting: framework for a SNA satellite system. Rev. Income Wealth.

[b0020] Braat L.C., de Groot R. (2012). The ecosystem services agenda: Bridging the worlds of natural science and economics, conservation and development, and public and private policy. Ecosyst. Serv..

[b0025] Britz W., Witzke H.P. (2014). CAPRI Model Documentation 2014.

[b0030] Burkhard B., Maes J. (2017). Mapping Ecosystem Services.

[b9005] Burkhard B., Kroll F. (2012). Mapping ecosystem service supply, demand and budgets. Ecological Indicators.

[b0035] Busch M., La Notte A., Laporte V., Erhard M. (2012). Potentials of quantitative and qualitative approaches to assessing ecosystem services. Ecol. Ind..

[b0040] Costanza R., de Groot R., Braat L., Kubiszewski I., Fioramonti L., Sutton P., Farber S., Grasso M. (2017). Twenty years of ecosystem services: How far have we come and how far do we still need to go?. Ecosyst. Serv..

[b0045] Daily G.C., Matson P.A. (2008). Ecosystem services: from theory to implementation. Proc. Natl. Acad. Sci..

[b0050] European Commission, European Environment Agency (2016). Report on Phase 1 of the Knowledge Innovation Project on an Integrated System of Natural Capital and Ecosystem Services Accounting in the EU (KIP-INCA Phase 1 Report).

[b0055] European Commission, International Monetary Fund, Organisation for Economic Co-operation and Development, United Nations, World Bank (2009). System of National Accounts 2008.

[b0060] European Commission, International Monetary Fund, Organisation for Economic Co-operation and Development, United Nations, World Bank (1993). System of National Accounts 1993. Brussels/Luxembourg, New York, Paris, Washington, DC.

[b0065] Fisher B., Turner R.K., Morling P. (2009). Defining and classifying ecosystem services for decision making. Ecol. Econ..

[b0070] FAO (2003). Cross-sectoral Policy Impacts between Forestry and Other Sectors.

[b0075] FAO (2018). System of Environmental-Economic Accounting for Agriculture, Forestry and Fisheries: SEEA AFF.

[b0080] Grizzetti B., Bouraoui F., Aloe A. (2012). Changes of nitrogen and phosphorus loads to European seas. Glob. Change Biol..

[b0085] Haines-Young, R., Potschin, M.B. (2018). Common International Classification of Ecosystem Services (CICES) V5.1 and Guidance on the Application of the Revised Structure.

[b0090] Hein L., Bagstad K., Edens B., Obst C., de Jong R., Lesschen J.P. (2016). Defining ecosystem assets for natural capital accounting. PLoS ONE.

[b0095] Jones L., Norton L., Austin Z., Browne A.L., Donovan D., Emmett B.A., Grabowski Z.J., Howard D.C., Jones J.P.G., Kenter J.O., Manley W., Morris C., Robinson D.A., Short C., Siriwardena G.M., Stevens C.J., Storkey J., Waters R.D., Willis G.F. (2016). Stocks and flows of natural and human-derived capital in ecosystem services. Land Use Policy.

[b9000] La Notte A.S., Vallecillo S., Maes J. (2019). Capacity as “virtual stock” in ecosystem services accounting. Ecological Indicators.

[b0100] La Notte A., D’Amato D., Mäkinen H., Paracchini M.L., Liquete C., Egoh B., Geneletti D., Crossman N.D. (2017). Ecosystem services classification: a systems ecology perspective of the cascade framework. Ecol. Ind..

[b0105] La Notte A., Maes J., Dalmazzone S., Crossman N.D., Grizzetti B., Bidoglio G. (2017). Physical and monetary ecosystem service accounts for Europe: a case study for in-stream nitrogen retention. Ecosyst. Serv..

[b0120] La Notte A., Vallecillo S., Polce C., Zulian G., Maes J. (2017). Implementing an EU System of Accounting for Ecosystems and Their Services: Initial Proposals for the Implementation of Ecosystem Services Accounts.

[b0110] La Notte A., Maes J., Thieu V., Bouraoui F., Masi F. (2012). Biophysical Assessment and Monetary Valuation of Ecosystem Services: Scenario Analysis for the Case of Water Purification in Europe.

[b0115] La Notte A., Marques A. (2017). The role of enabling actors in ecosystem service accounting. One Ecosystem.

[b0125] Lange G.-M. (1999). Forum: how to make progress toward integrating biophysical and economic assessments. Ecol. Econ..

[b0130] Leip A., Marchi G., Koeble R., Kempen M., Britz W., Li C. (2008). Linking an economic model for European agriculture with a mechanistic model to estimate nitrogen and carbon losses from arable soils in Europe. Biogeosciences.

[b0135] Lutz E. (1993). Toward Improved Accounting for the Environment.

[b0140] Maes J., Teller A., Erhard M., Grizzetti B., Barredo J.I., Paracchini M.L., Condé S., Somma F., Orgiazzi A., Jones A., Zulian G., Vallecilo S., Petersen J.E., Marquardt D., Kovacevic V., Abdul Malak D., Marin A.I., Czúcz B., Mauri A., Loffler P., Bastrup-Birk, Biala K., Christiansen T., Werner B. (2018). Mapping and Assessment of Ecosystems and Their Services: An Analytical Framework for Ecosystem Condition.

[b0145] Maes J., Egoh B., Willemen L., Liquete C., Vihervaara P., Schägner J.P., Grizzetti B., Drakou E.G., La Notte A., Zulian G., Bouraoui F., Luisa Paracchini M., Braat L., Bidoglio G. (2012). Mapping ecosystem services for policy support and decision making in the European Union. Ecosyst. Serv..

[b0150] Mäler K.-G., Aniyar S., Jansson Å. (2008). Accounting for ecosystem services as a way to understand the requirements for sustainable development. Proc. Natl. Acad. Sci..

[b0155] Millennium Ecosystem Assessment (2005). Ecosystems and Human Well-being.

[b0160] Obst C., Hein L., Edens B. (2016). National accounting and the valuation of ecosystem assets and their services. Environ. Resour. Econ..

[b0165] Pèrez-Soba M., Elbersen B., Kempen M., Braat L., Staristky I., Van der Wijngaart R., Kaphengst T., Andersen E., Germer L., Smith L., Rega C., Paracchini M.L. (2015). Agricultural Biomass as Provisioning Ecosystem Service: Quantification of Energy Flows.

[b0170] Polce C., Termansen M., Aguirre-Gutiérrez J., Boatman N.D., Budge G.E., Crowe A., Garratt M.P., Pietravalle S., Potts S.G., Ramirez J.A., Somerwill K.E., Biesmeijer J.C. (2013). Species distribution models for crop pollination: a modelling framework applied to Great Britain. PLoS ONE.

[b0175] Potschin M., Haines-Young R., Fish R., Turner R.K. (2016). Routledge Handbook of Ecosystem Services.

[b0180] Remme R.P., Edens B., Schröter M., Hein L. (2015). Monetary accounting of ecosystem services: a test case for Limburg province, the Netherlands. Ecol. Econ..

[b0185] Schröter M., Barton D.N. (2014). Accounting for capacity and flow of ecosystem services: a conceptual model and a case study for Telemark, Norway. Ecol. Ind..

[b0190] Science for Environment Policy (2017). Taking Stock: Progress in Natural Capital Accounting, In-depth Report 16 Produced for the European Commission.

[b0195] Serna-Chavez H.M., Schulp C.J.E., van Bodegom P.M., Bouten W., Verburg P.H., Davidson M.D. (2014). A quantitative framework for assessing spatial flows of ecosystem services. Ecol. Ind..

[b0200] Syrbe R.-U., Walz U. (2012). Spatial indicators for the assessment of ecosystem services: providing, benefiting and connecting areas and landscape metrics. Ecol. Ind..

[b0205] TEEB (2010). The Economics of Ecosystems and Biodiversity Ecological and Economic Foundations.

[b0210] United Nations (1993). Integrated Environmental and Economic Accounting, Interim Version. United Nations, New York.

[b0215] United Nations (2017). Technical Recommendations in Support of the System of Environmental-Economic Accounting 2012 – Experimental Ecosystem Accounting.

[b0220] United Nations, European Union, Food and Agriculture Organization of the United Nations, International Monetary Fund, Organisation for Economic Co-operation and Development, World Bank (2014). System of Environmental-Economic Accounting 2012 — Central Framework.

[b0225] United Nations, European Union, Food and Agriculture Organization of the United Nations, Organisation for Economic Co-operation and Development, Group, World Bank (2014). System of Environmental-Economic Accounting 2012—Experimental Ecosystem Accounting.

[b0230] Vallecillo S., La Notte A., Polce C., Zulian G., Alexandris N., Ferrini S., Maes J. (2018). Ecosystem Services Accounting: Part I – Outdoor Recreation and Crop Pollination.

[b0235] Villa F., Bagstad K.J., Voigt B., Johnson G.W., Portela R., Honzák M., Batker D. (2014). A methodology for adaptable and robust ecosystem services assessment. PLoS ONE.

[b0240] Wealth Accounting and the Valuation of Ecosystem Service (2012). Moving Beyond GDP: How to Factor Natural Capital into Economic Decision Making.

[b0245] Zulian G., Maes J., Paracchini M. (2013). Linking land cover data and crop yields for mapping and assessment of pollination services in Europe. Land.

[b0250] Zulian G., Paracchini M.L., Maes J., Liquete M. (2013). ESTIMAP: Ecosystem Services Mapping at European Scale.

